# Loss of Close Relationships and Loss of Religious Belonging as Cumulative Ostracism: From Social Death to Social Resurrection

**DOI:** 10.3390/bs10060099

**Published:** 2020-06-10

**Authors:** Adriano Zamperini, Marialuisa Menegatto, Miriam Mostacchi, Simone Barbagallo, Ines Testoni

**Affiliations:** FISPPA Department, University of Padova, 35131 Padova, Italy; marialuisa.menegatto@unipd.it (M.M.); setti.mostacchi@libero.it (M.M.); simo.barbagallo@libero.it (S.B.); ines.testoni@unipd.it (I.T.)

**Keywords:** exclusion, sources of ostracism, consequences of ostracism, account-making, everyday life, social trauma, marriage, divorce, religious group, religious community

## Abstract

Background: Ostracism is a negative experience that has been studied primarily in laboratory settings. This study extends current research by investigating ostracism in daily life, analyzing the transition from social death to social resurrection of Catholic partners who suffered ostracism both in the couple (with the consequent divorce) and in the religious community they belong to (ban from religious practices). Therefore, we introduce the notion of ‘cumulative ostracism’. Method: Data are composed of the biographical narratives of n = 25 participants in a religious experience group in a period of 7 months. A narrative analysis was used within the framework of the temporal need–threat model. Results: Participants’ narratives are consistent with the temporal need–threat model. The ostracism experience, both in the couple and in the religious group, is characterized by the sequence: immediate stage, coping stage and resignation stage. Moreover, the cumulative ostracism suffered by the participants presents an analogy with the cumulative trauma that occurs in intra-family abuse. Social resurrection occurs through the encounter with a new religious group that allows ostracized people to experience a Catholic religious affiliation again. Conclusions: Our findings reflect the existing theory and add to the existing laboratory research by capturing ostracism-unique dynamics in real life.

## 1. Introduction

The scientific literature has systematically shown that being ostracized constitutes an experience of psychological pain. In short, ostracism arouses social suffering because four basic needs are threatened, according to Williams’ need–threat model [1]. First, ostracism threatens our need to belong; this need for frequent and enjoyable interactions [2] is believed to have evolved because inclusion in a group meant greater chances of survival and reproductive benefits for our ancestors in hostile environments [3]. Second, ostracism threatens the need to maintain high self-esteem because it carries with it the implicit or explicit accusation of having done something wrong. Third, ostracism can threaten the need for control over interactions with others. Fourth, ostracism can threaten the need to maintain our belief in leading a meaningful existence [4,5,6,7] because it could remind us of our fragile and temporary existence, and even of our own death [8,9]. One meta-analysis found that ostracism induced in experimental settings is a relational condition that undermines these four needs [10]. Furthermore, ostracism has been shown to induce a state of insignificance and to increase cognitive accessibility to thoughts about death [11].

Not only can ostracism remind people of their own death, but it is also metaphorically defined as ‘social death’ [12,13]. The term “social death” is commonly used by scholars in several different disciplines and first entered social science vocabulary referring to the social processes according to which medical personnel give different social value to patients, thus leading to different medical efforts [14]. This implicates that awareness of a person’s dying determines social interaction [15]: this idea is developed on the ground of the concept of mortification of self by Erving Goffman [16], who describes the series of humiliations undermining a person’s social identity in a psychiatric asylum. Usually, researchers referring to social death apply the concept to state that a person or a group has experienced an extreme and profound loss. The term is used in sociology [17]; in intercultural studies, referring to the spatial segregation and loss of citizenship of migrants and asylum seekers in the arrival country [18]; in the history of slavery, to analyze the interaction of power dynamics and the loss of cultural heritage and of cross-generational connections [19], a concept that is used also in the studies of genocide [20]; in the social psychology of groups, to deal with cooperative behavior and the bad apple effect [21]; in existential psychotherapy [22].

But is it true that ostracism equals death? In order to answer this question, Hales used four components of the concept of death identified in developmental psychology literature: (1) universality—all living things die, without exception; (2) causality—death is caused by some things but not by other things; (3) total non-functionality—all bodily and mental functions are completely terminated at death; (4) irreversibility—death is final and cannot be undone [12]. Furthermore, if social death is an appropriate metaphor for ostracism, four consequences emerge: (1) social invincibility, (2) social autopsy, (3) social necromancy, and (4) social resurrection [12].

However, in the case of ostracism, death is not always irreversible: most episodes of ostracism can undergo an inverse process that allows the ostracized to experience social inclusion again (social resurrection). This especially occurs when ostracism is used as a tool to correct attitudes and/or behaviors—that is, exile works as a warning to force change [23]. Indeed, research shows that after having suffered ostracism, people engage more in prosocial behaviors aimed at readmission to the group and/or community [24]. Commitment to satisfy the mortified needs of belonging, self-esteem, control and significant existence certainly does not necessarily imply a successful outcome. Struggling with social death to regain a social life is certainly something extraordinary and not everyone has a second chance to reconnect in interpersonal relationships or re-enter social groups. Undoubtedly, it can be relatively easy to gain readmission in the face of mild and temporary forms of ostracism. In contrast, reaching readmission after severe cases and after a long period of ostracism is much more difficult; it is a social difficulty for ostracized people and a scientific difficulty for researchers studying ostracism. Much of the existing research, in part for ethical reasons, is mainly based on laboratory research that analyses mild and short forms of ostracism. Role-playing games and simulations are often used to induce ostracism among strangers. These are without a previous relational history and without a future, therefore creating situations that have limited emotional involvement [10,25]. One study tried to investigate ostracism in real life [26,27]. The results showed that people reported more frequent episodes of ostracism with strangers, whereas being the target of ostracism by family and romantic partners was less frequently mentioned [28]. However, research on family ostracism has shown that it is not rare to ostracize a member of the family, and that the severity of the phenomena of ostracism occurs in their duration of months or even years [29], causing devastating effects when the prolonged duration seems to infinitely dilate the psychological pain [30]. That being said, compared to ostracism experiences with strangers, family ostracism is a much more painful experience. It is thus clear that the phenomenon of ostracism must be analyzed considering the factors of both time and severity.

The time factor in ostracism practices is addressed by the temporal need–threat model [1,31], which includes three successive phases. In Phase I, an individual is excluded—here, being ignored and refused arouses a state of alertness and imminent danger [32]. In the immediate term, the individual’s response to the threat involves pain and anguish, considered as adaptive reactions for survival in which sadness and anger prevail. In Phase II, the ostracized person engages in the work of interpretation mediated by his or her cognitive processes in an attempt to make attributions and assessments on the painful episode, engaging in prosocial behaviors to recover the need for belonging, self-esteem, understanding, sharing and trust [23,33]. In contrast, if the trend is aimed at strengthening the need for efficacy, meaningful existence, control and recognition, an individual may respond with anti-social behavior. Should his or her efforts to overcome exclusion fail, and exposure to ostracism becomes a chronic state, the person’s level of negative emotions increases, and his or her coping resources are weakened, placing the individual in Phase III. This is characterized by feelings of depression, alienation, unworthiness and impotence [34].

The severity factor certainly concerns the characteristics of the ostracizing act (e.g., being excluded for a few minutes from an online video game has a smaller psychological impact than being considered the black sheep of a peer group at school), but it lends itself to further considerations. In fact, if social death is configured as a loss that compromises the basic needs of a human being, this loss is not necessarily a singular loss. According to Norwood [35], social death is a series of losses that, acting cumulatively, can lead to an individual’s serious disconnection from social life, throwing him or her into existential anxiety. Trauma studies [36,37,38,39,40] have highlighted the possibility that different traumatic events affecting a single person—child or adult—occur in the same time frame.

As Cloitre et al. [41] argued, the literature on interpersonal violence suggests that counting the types of exposure to traumatic events per individual is a solid predictor of negative psychosocial outcomes, suggesting that there may be something particularly harmful in experiencing multiple forms of interpersonal victimization. In summary, the literature indicates that people with a history of exposure to different types of traumatic events are more likely to experience a broader symptomatology than those with more circumscribed traumatic event histories. Similar to what trauma studies indicate, the present researchers believe it is possible to extend these considerations to suffering from social death. In real life, the situation of people forced to undergo repeated refusals in different social areas is not uncommon. Just as the symptomatology of people exposed to numerous traumatic events is serious and complex, the possibility of a social resurrection will be much more difficult in the presence of a series of episodes of ostracism that add to each other in a cumulative way. Therefore, in this article, the researchers introduce the notion of ‘cumulative ostracism’ to describe the repeated action of different forms of ostracism implemented by different sources that affect a single target. Ultimately, to understand individuals’ efforts to free themselves from social death and reach social resurrection, the researchers argue it is necessary to consider both the time factor (temporal model) and the severity factor (additive model).

If, on the whole, what is known about serious and long-term forms of ostracism within close relationships (e.g., in a family) derives mostly from anecdotal reports, diaries and/or novels, the present research intends to contribute to the knowledge of ostracism in real life [42]. The researchers analyze the transition from social death to social resurrection of Catholic partners who have suffered ostracism both in the couple (with the consequent divorce) and in the religious community to which they belong (social isolation and banishment from religious practices).

## 2. Materials and Method

### 2.1. First Stage of the Research

#### 2.1.1. Setting

Like much of Western Europe, Italy is characterized by a growing feeling of religious indifference [43]; however, for some people, religion still represents an important aspect of personal growth and social guidance [44,45]. In addition, many ecclesial movements and groups constitute networks of relationships in which one experiences inclusive communities—being part of them is an aspect that structures subjective existential meaning as well as individual and social well-being.

For some practicing Catholics, because of their commitment to the conjugal bond celebrated as a sacrament within the Church, the dissolution of said bond represents a psychological pain that equates to a breakdown of the relationship with the partner. The state of discomfort they experience increases in the context of the religious community to which they belong because of the clear feeling of being wrong.

In light of this socio-cultural background, the present research took place at the Family Spirituality Center *Casa della Tenerezza* (House of Tenderness) (Umbria, Italy), founded by the Catholic priest and theologian Carlo Rocchetta in 2003 as a pastoral care project for couples and families. It is particularly focused on spouses having difficulty because they have separated, divorced, remarried or are facing any type of irregular coexistence with respect to the principles of the Catholic Church.

#### 2.1.2. Field Visits

Initially, the researchers contacted the House of Tenderness by telephone to explain their research purposes. They subsequently conducted two introductory informative talks with the coordinators of the House of Tenderness (a Franciscan religious group called the Order of Friars Minor as well as a married couple who helps to run the House of Tenderness). Once the association gave its consent to the research, one of the researchers participated in the periodic activities the House of Tenderness carried out, including informal meetings and study days aimed at divorced people, priests, pastoral care workers, professionals and social workers who dealt with family support after separation.

This first phase, which lasted three months, allowed them to familiarize themselves with the research field and understand the purpose and organization of the center, reaching the specific area of the investigation in the ‘Berit’ religious experience group. The term ‘Berit’ derives from Hebrew and refers to the ‘covenant’ between Yahweh and humanity. The reference to the covenant intends to underline that, although the bond/covenant with a spouse has been broken, the Lord does not stop wanting to make covenants with everyone and continues to seek relationships with everyone.

### 2.2. Second Stage of the Research

#### 2.2.1. Participants and Procedure

The Berit religious experience group has 25 participants (12 men and 13 women, age range 35–60, 17 with a high school diploma, and 8 with a post-secondary degree); it is coordinated by three co-conductors (one religious conductor and a married couple). Since participation is free, not all of the participants are always present at every session; however, for each session, the minimum number of participants is 20. The number of participants is in line with the usual standards of qualitative research, since research has shown that the collection of qualitative data on more than a certain number of people interviewed does not produce any addition of knowledge as it generates a data saturation effect [46].

The participants are all separated or divorced (marriage duration range 2–20 years, with at least one child born into the marriage) and participate in the group alone—that is, without the presence of the ex-wife or ex-husband. On average, sessions take place once a month, and each session lasts for three hours. The co-conductors of the group presented the aims of the research to the participants. Once they obtained their consent, one of the researchers joined the group as a participating observer. For each session, the researcher wrote brief ethnographic notes on themes that emerged during the session. The research took place from January 2019 to July 2019. During this period, the researcher participated in seven sessions. The first group session started with the presentations: the participants told who they are, their stories, and what led them to join the group. The research team member was introduced by the conductors and then told the audience about the objectives of this study. So, the meeting went on as usual. The members discussed biographical and religious themes, reading and commenting on some passages from the Bible. Up to and including the fifth meeting, the group continued to carry out its usual discussion activities. The last two sessions, on the proposal of the member of the research group, have entirely focused on the theme of ostracism on the basis of the short ethnographic notes collected and the personal experiences of ostracism spontaneously told by the participants in the first five meetings. This study followed the American Psychological Association Ethical Principles of Psychologists and Code of Conduct and the principles of the Declaration of Helsinki. Each participant signed an informed consent form.

This study was approved by the Ethical Committee of the University of Padova (Ethical’s Committee approval protocol: E278EC03606834974A43F6BD458DD6E8; approval date: 10 April 2017).

#### 2.2.2. Method

The research adopted a qualitative approach [47] following the CoreQ checklist [48]. The process was divided into six main phases: preparatory organization, generation of categories or themes, data coding, testing emergent ideas, searching for alternative explanations and writing up the report [49]. The narrative corpus of the analysis was composed of the biographical narratives [50] of the participants in the religious experience group for the period considered (January 2019–July 2019).

The biographical narratives collected during the seven sessions were audio-recorded and transcribed verbatim. All the transcripts were analyzed using a thematic analysis approach [51] with the support of the software Atlas.ti [52]. The coders read Williams’ [9,31,32,53] papers on ostracism in order to effectively code the data. The code sheet they used required each rater to code six elements about the biographical narratives: the source of ostracism (marriage, religious group), the link between the sources of ostracism (marriage, religious group), the stages of the temporal need–threat model (immediate stage, coping stage, resignation stage), the impact of ostracism on psychological needs (belonging, self-esteem, self-control, sense of meaningful existence), the consequences of ostracism (separation/divorce, isolation from a religious group) and the restoration of inclusion (close relationships, religious group).

To validate the coding, the coders—two of the researchers—worked independently to categorize the phrases and sentences. After coding independently, the two coders and the research leader met and discussed any discrepancies. The coders achieved a good level of interrater agreement (80% or greater) for each code. When disagreements occurred, the research leader and another member of the research group decided which code was more appropriate. During this process, the codes were continually refined. The researchers acknowledge the possibility for bias because the two coders were also researchers. However, having extensive expertise in both coding and theoretical perspectives on ostracism helped them to explore unexpected themes in the biographical narratives. Finally, all members of the research group often discussed coding to ensure everyone was clear about the procedure and how to explain it.

The researchers named and classified through open coding the textual data produced by the participants. Then the text segments have been compared and similar ones have been grouped together and endowed with the same conceptual label for themes and subthemes. In addition, with the axial coding, the parts of the data identified and separated have been brought together in order to establish connections between themes and subthemes. In this way, the complexity of the object of investigation can be reported in the form of a figure. The texts provide clues on how themes and subthemes relate or not. Working with data from real situations, relationships among them may not always be so obvious. The links between themes and subthemes can be subtle or implicit. Therefore, through the two group sessions entirely focused on ostracism, making use of questions such as “where, how, when, why and who”, the relationships between themes and subthemes have been better identified and clarified. The thematic areas form the basis for the structure of this article—extracts of which (translated in English by a native speaker) are used to illustrate key results.

## 3. Results

### 3.1. Thematic Analysis: List of Themes and SubThemes

Table 1 and Table 2 list the themes (first column) and the subthemes (second column) that emerged about ostracism in marriage and ostracism in religious group. The instances of occurrence (third column) refer to the sum of the quotations (the verbatim evidence given by the participants) for each subtheme. The occurrences indicated in the third column exclusively refer to the six elements about the biographical narratives listed in the Method section (further textual data not relevant to these research elements have not been considered). As each subtheme could be mentioned several times by a single participant during the seven group sessions, the percentage across participants (fourth column) describes the percentage of participants who, during the seven sessions, produced at least once textual data connected to the specific subtheme. It is important to underline that the frequencies in Table 1 and Table 2 exclusively refer to textual segments produced by verbal communication by the participants to the group session. This means, for example, that, when a participant said to have felt inadequate (ostracism in religious group, theme Resignation stage, subtheme Inadequacy) and another participant agreed with an expression as “So did I”, the researchers counted this expression relating to the subtheme Inadequacy, while, if the agreement was produced through the non-verbal channel, for example nodding, the researchers did not count this physical indicator in the subtheme Inadequacy. As non-verbal communication has a different level of ambiguity, thus making the codification process difficult, and since there was only one member of the research team present during the sessions, only the verbal material was codified in order to avoid biases.

### 3.2. Thematic Analysis: Timeline of the Themes

**Figure 1:**
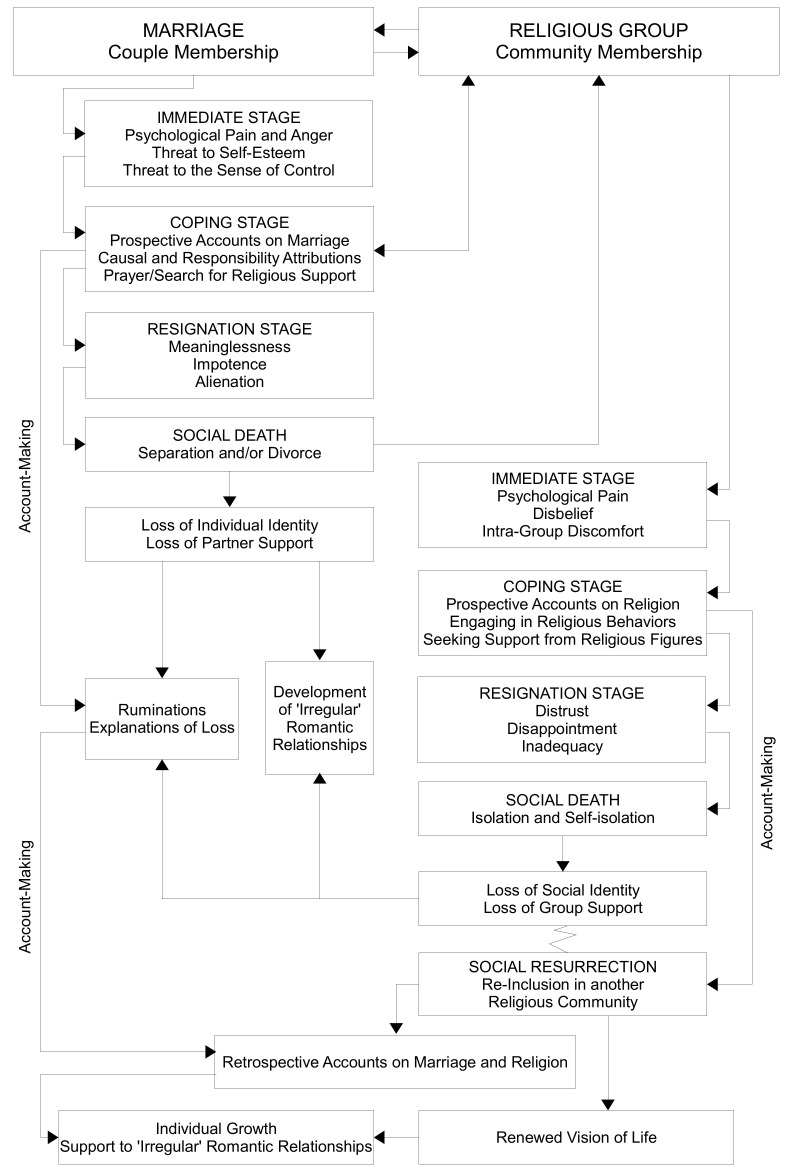
Timeline tree of cumulative ostracism.

Figure 1 shows ostracism within marriage in the left column and ostracism within the religious community in the right column. For each form of ostracism, the researchers organized the psychological processes as foreseen by the different stages of the temporal need–threat model. They developed a timeline tree [54] to illustrate the transactions between the elements of the two forms of ostracism that occurred over time. The elements of the transactions are represented by boxes.

Ostracism within marriage is the emerging signal of a couple crisis that forces the partner who suffers it to question himself in terms of the marriage bond, in light of a marriage sanctioned by a religious contract that would prohibit its dissolution. In fact, in the coping stage, a process of mutual influence emerges between subjective resources and resources of the religious group in order to try to cope with the situation that has arisen. As long as the ostracized partner manages to keep the conjugal bond “alive” despite the painful difficulties, he/she can count on the support of the religious group. When he/she capitulates, resigning himself/herself to the breakdown of the marriage bond, he/she feels the eruption of relational death in his/her life and a catastrophic loss of identity. At the same time, the temporal organization of the phenomenon of ostracism has allowed the researchers to highlight how the first social death, which corresponds to separation, becomes the cause of a new process of ostracization that culminates in a second social death—isolation and exclusion from the religious community. In fact, since marital bond and religious belonging are in this case closely interconnected, the end of the former opens up to the possibility of no longer being a member of the religious group, being even moved away by its representatives. Here too, the three stages of the temporal need–threat model are clearly visible: the person ostracized by the religious group initially appears incredulous, tries to commit himself/herself to maintaining membership but in the end he/she has to resign himself/herself, he/she is moved away and in part moves away on his/her own from the religious group. The loss of social identity and group support is added to the loss of personal identity and partner support. 

In the timeline tree, it is possible to note that social, marriage and religious group death is not necessarily a definitive closure. In fact, ostracized partners continue to present an account-making activity relating to the sense of emotional loss and the loss of religious affiliation. While attempts to build new romantic relationships by seeking alternative relational resources are observed, the same attempts are not found with regard to the religious group. The zigzag line that connects the “loss of social identity/loss of group support” box to the “social resurrection” box indicates that the route was random, fortuitous and unplanned. Anyhow, the dimension where there is a real resurrection concerns re-inclusion in a new religious community, which leads to a renewed vision of life that is capable of supporting and promoting—at least in part—the individual identity of those who remain single as well as the efforts of those who try to develop new ‘irregular’ (according to Church doctrine) romantic relationships. Even in this phase of social resurrection, the account-making activity does not cease: as a further demonstration of the close connection between the dimensions of marriage and the Catholic group, the account-making process concerns both couple life and life in a religious community. A forward movement in the life of each participant looking behind them, in a process of re-storying of one’s life.

### 3.3. Thematic Analysis: The Contents

#### 3.3.1. From Marriage to Social Death

The participants narratively re-evoked the phenomenon of ostracism within the marriage crisis following the stages of the temporal need–threat model. Open rejection and silent treatment were the most frequently re-evoked ostracizing modalities; they were associated with feelings of devaluation (attack on self-esteem), loss of control (relating to the couple interactions and the children’s education) and intense anger. Although ostracism hurt and caused negative emotions (immediate stage), as long as the partner perceived that he or she had some chance of regaining inclusion in the couple relationship, he or she engaged in a strenuous relational struggle (coping stage). He or she reflected on the significance of the ostracizing experience he or she had undergone, operated attribution processes to identify causes and responsibilities, constructed an account for interpreting his or her relationship experience and activated all available resources to avoid the dissolution of the marriage. Being Catholic partners, among the strategies to cope with the marriage crisis, they also used prayer and religious practices to try to strengthen themselves—to experience belonging to the religious community. The participants used all the available means to resist exclusion, up to exhausting their coping resources and accepting their ostracized condition (the resignation stage):

… in the end, I couldn’t take it anymore, I had tried everything…Let’s separate! If this is the only thing he wanted…in the end, I couldn’t take it anymore…

The experience of separation as social death is well represented by the following testimony:

When I realised that I would have to separate, I thought, “I’ve lost”, because the desire to have a family had always been great. At that moment, I experienced a mixture of feelings. You would throw everything away…You feel like a person who has lost. You feel knocked out because you devoted all of yourself to that project…you feel like a loser. You are in a land similar to a garbage dump, where you throw everything away, even the beautiful things…even my daughter—she too seemed to me the wrong thing.

The breakdown of the marriage bond represents a stressful and painful event characterized by the loss of the partner and by a strong identity instability (itself another loss): the loss of existential balance. The various psychological difficulties (including self-blame, anguish and a sense of helplessness and insignificance) made the participants feel like victims of separation. Temporarily, after losing membership to the couple, the separated partners had to face the threats related to their religious affiliation.

#### 3.3.2. Social Death in the Religious Community

Religious membership in this moment of biographical change did not appear to be an immediate resource for coping with the situation of relationship loss—rather, it was an aggravating factor. As one woman described,

(We were) a “model” couple for all the other couples of friends and for the group of people engaged in parish activities…We had a crisis, we separated…and this has multiplied the crisis because everyone had high expectations, because they thought that the Lord would have protected us from any crisis…everyone was scandalised by our failure, even we were, and this has complicated things…

In the following participants’ narratives, their relationship instability consolidated and became associated with a religious instability that quickly began to manifest (immediate stage). The separated partners experienced initial refusals:

I’ve been through a lot…In the parish group, they were gossiping and talking badly about me behind my back…I phoned the priest for the Easter blessing, I asked if he would come at least for the children, but nothing. Then I realised that he didn’t come because I was separated.

After the separation, I had many problems: feelings of guilt, the fear of having done everything wrong, of not having worked hard enough to try to save the marriage. So, I felt the need to speak with a priest, and I went to confession. Well, I was thrown out of the confessional, the priest said to me: “No, you cannot approach this sacrament because you are separated”.

The first thing I heard (from the parish priest) was: “Well, however…with regard to the sacraments, it’s a problem…” Then, on another occasion, he said to me, “You can no longer read in Church”. And when I was feeling bad, I needed to confide and to talk, the parish priest did not even receive me. The matter of the belonging to the Church was however a wound…because I felt as though I was no longer part of the Church.

The experience all the participants shared was that of refusal and estrangement from the respective religious communities to which they belonged following their separation and/or divorce. Despite repeated attempts to remain within the religious group (coping stage), their resources ran out, and resignation took over (resignation stage). As a final consequence of this second experience of social death, a sort of ‘self-ostracism’ took place with respect to the Church in general: the refusal suffered in their own religious community lead the participants to avoid making further attempts toward other religious groups, isolating themselves even more:

I have been away from the Church for many years. For many years, I have avoided participating in religious groups engaged in the parish…a bridge with God had been interrupted…Surely each of us has been moved away, but we moved away too.

Self-estrangement originated from feelings of inadequacy when participating in the life of the ecclesial community or religious movements, increasing the feeling of discomfort in those who remained single and were still involved in a process of rumination over what went wrong in their marriages. The inadequacy is even more accentuated in the narratives of participants who were separated and later sought relational alternatives by developing new romantic relationships. According to Church doctrine, participating in an ‘irregular couple’ is now part of the sin of divorce—a ‘double inadequacy’:

I thought the Church was basically closed, I am a sinner: I am a separated person, I have a romance with another woman…How can I participate in the sacraments?

#### 3.3.3. Toward Social Resurrection

The participants described meeting with the House of Tenderness, itself a religious community, in terms of surprise and distrust:

A friend of mine suggested going to talk to the friar (of the House of Tenderness)…He convinced me, and I accepted … I got ready and then thought of another condemnation…But instead, I found a different perspective…After telling my story, he (the friar) said to me: “Sorry, ehm…but they did everything wrong with you”. I got curious…And he explained to me what it means to be part of the Church as baptized…Then he invited me to the House of Tenderness. I expected answers from the first meeting, like who goes to the psychologist, right? Instead, no one said to me, “You have to do this, you have to do that”. Then we read the Bible, and together, we listened to what it had to say to us today, here in our lives…

The language the participants used tells of their condition as Catholics without religious citizenship, without a parish of reference, amazed and happy to have found a home that welcomes them:

Here, we are all separated. Seeing other people like me face to face, people who have lived the same things…and this approach to religion in which I found doors open…here I was given the opportunity to see myself as something else besides what I thought of myself…because by then I had considered as mine the diagnosis made to me by a friar who had deliberately moved me off from a church, saying to me, “You are separated, what are you doing here? Either you take all the rules of what you say you love, or you don’t take any. If you get separated, you can’t get close to religion.” And I had made this idea my own…I was wrong…I was separated and I could not participate in religious life. I felt a weight, didn’t I? It seemed I was carrying a cross, however—and everywhere. [Instead], here you feel loved as a son of God, accepted, you exist, and you are a person worthy of every respect beyond the sin that you may have committed.

… here, I found the welcome of people who have lived my own experience, who can understand me, who can empathise with me, who know how to listen to you in a different way from a friend, a brother, a parent…Everyone keeps his own pain with himself because every story and every person is independent, but we are all on the same wavelength, we understand what pain we are talking about…And this is an important thing.

A woman summarized the cycle as ‘death within marriage—death within the religious community—resurrection’:

I have lost a husband, I have lost and regained the Faith, and I have a new community.

Resurrection involves obtaining a new vision of life; it contributes to the recovery and personal growth of the participants who were separated and for those who became part of a new couple, helping them to manage the condition of being ‘doubly irregular’ in the eyes of most of the Catholic world, also encouraging people to become more involved in prosocial behavior.

## 4. Discussion

When studying ostracism not in laboratory situations but in daily life, a choice is generally made between one of the following two strategies: using large questionnaires or adopting a repeated measures method [26,27]. In this study, the researchers tried to broaden their investigation strategies into separated and/or divorced Catholics’ daily lives by exploring their experiences of ostracism through the biographical narratives they produced during their participation in the sessions of a Catholic religious experience group. The participants underwent a transformative journey that engaged them in mental processes of interpretation, evaluation and attribution of new meanings to their life experiences.

Two phenomena of ostracism emerged from the participants’ biographical narratives: ostracism during marriage and ostracism in the religious community. Ostracism in marriage leads to the separation of the couple (the first episode of social death) and causes ostracism in the religious group they belong to, which in turn causes rejection and removal from the group (the second episode of social death).

This cumulative ostracism generates great psychological distress in the participants, who are forced to suffer a series of losses one after the other: within the couple, loss of the individual identity of husband and wife and loss of partner support; within the religious group, loss of the social identity of Catholic affiliation and loss of group support—for some, even loss of faith. Social resurrection occurs through the encounter with a new religious group that allows ostracized people, despite their condition of being separated or in new ‘irregular’ (as per the doctrine of the Catholic Church) romantic relationships to experience a Catholic religious affiliation again. Furthermore, feeling re-included in one’s own religious community and being able to experience a sense of greater connection with God increases personal well-being and promotes sympathetic emotions [55].

The participants’ narratives are consistent with the temporal need–threat model [31]: the ostracism experience, both in the couple and in the religious group, is characterized by the sequence of immediate stage, coping stage and resignation stage. As for ostracism in marriage, the use of religion emerges in the coping stage as an attempt to cope with the situation. While the literature identifies resources in prayer and religious commitment that can promote recovery from short-term ostracism [56]—that is, after suffering a social death—in this study, the resources were also used during the coping stage. Since the participants were affiliated with local religious groups, and the marriages were lived within the doctrine of the Catholic Church, the partners evidently sought support in the religious group of reference in order to solve the problems they were experiencing as a couple and resorted to prayer to fortify themselves. Therefore, religion was not only at the service of recovery but also of coping.

The separation of the couple triggers the subsequent phenomenon of religious ostracism, and the partners’ immediate emotional reaction (immediate stage) is of psychological pain associated with a sense of disbelief (while in the immediate stage of a couple’s ostracism, the association is between psychological pain and anger). The partners probably expect, especially in a difficult biographical moment (the breakdown of the marriage), greater solidarity and pastoral care. Although the Catholic Church should keep everyone beneath its roof, they are instead discarded and excluded—not only by friends and members of the religious group they belong to, but also by priests. Although they strive to regain connection in the group during the coping stage, in the end, they are forced to give up, concluding that they are ‘inadequate’ for the Catholic religion. They are marked by a stigma [57] that cannot be removed, as the mark of separation is indelible. In fact, stigma is a sign that exposes the individual to being discredited, and stigmatization is the process that occurs when a person has certain attributes that convey an identity that is devalued within a certain social context. Even those who seek to rebuild a love life fail to free themselves from discredit. Indeed, being separated and then (according to the Catholic Church) ‘irregular’ cohabitation accentuates their discomfort and sense of inadequacy.

If the need to belong, in addition to the motivational pattern of satiation, also seems to conform to the motivational model of substitution [2], when a social bond is broken, people should better recover if they form a new one, even if each individual life tends to present some particularly special relationships (like marital bonds) that are not easily substitutable. In our case, in addition to the special emotional bond of marriage, a second special bond is associated: belonging to the Catholic group. We have repeatedly stressed that this twofold belonging has a strong character of interdependence: Catholic Church, on the basis of the Gospel of Matthew (“Let not that which has been joined by God be parted by man.”), constantly recalls the indissolubility of marriage. The sense of disorientation resulting from the breakdown of the marriage pact is twofold: on the one hand what the Christian community of the faithful feels and on the other that of the spouses themselves who separate. As a consequence, often ex-spouses and communities of the faithful react by distancing each other, in the form of exclusion or self-exclusion. Such a loss cannot be compensated through a substitution: it is not a question of finding a new partner or not; the problem is that divorce remains an indelible stigma for the Catholic Church (ban from rebuilding a couple bond like the broken one). Therefore, research participants, even if they seek emotional compensation with a new partner, have the perception and are perceived as unsuitable for the Catholic religion. Of course, they can maintain a private dialogue with God but the life of the religious community, guided by the priest’s authority (which is crucial in Catholic religious practice), is forbidden to them. Only by finding a new Catholic religious community, tolerant of the divorced, they can embark on a path of social resurrection at the group level.

Furthermore, it has been amply demonstrated how belonging to certain social categories is relevant to the concept of self [58] and, therefore, memberships can influence the way in which individuals respond to ostracism. Scientific research [59] suggests that the theory of cognitive dissonance [60] may contribute to widening the explanation levels of the phenomena of ostracism, in particular to investigate the management of the discomfort felt by the sources of ostracism: how they manage their concept of themselves knowing that they have caused harm to others. Following this indication, the researchers believe that the theory of cognitive dissonance can also help to explain in a broader sense the profound discomfort experienced by the research participants, the subjective sense of inadequacy and self-estrangement from the religious group. In fact, having a self-concept as a practicing Catholic and having divorced (albeit forced by the irremediably damaged marriage situation) means dissonance between one’s self-concept and what has been done. The subsequent ostracism suffered by the religious group only confirms that the individual identity has changed forever. Since divorce (behavior) is no longer modifiable, it is cognition (one’s self-concept) that has undergone a change. Hence the belief of not being adequate for the Catholic religion and withdrawal from this world. Only the encounter with a new religious group allows to rethink and rebuild one’s self-concept as a Catholic.

Note that the account-making activity present during the coping stage does not terminate with the resignation stage and the relative social death. This has been observed for both marriage and religious ostracism; it differs from previous work concerning the temporal sequence of ostracism that was carried out in a laboratory setting [61]. Constructing an account represents a significant tool in the coping process since it allows one to reflect on what is happening, reaching some coherent understanding and explanation of a disruptive and excruciating event. Even if this activity occurs when there is still hope of being able to overcome ostracism and avoid breaking the relationship between the couple and the group, this work aimed at giving meaning to the experience over time, even after the breakdown. In particular, while account-making relating to the finished marriage was very active in those who remained single, it lost salience for those who were engaged in new romantic relationships. When someone is separated and left alone, there is a strong drive to try to understand why things went wrong [62] and to come up with some form of reassurance that will protect the person from the fear of repeating the same experience in the future. While account-making seems to lose importance for those who are trying to develop new emotional relationships, it returns as a psychological process that engages the individual in the phase of affiliating with a new religious group. Since the couple life and the community life of these participants are oriented by the Catholic religion, and religion becomes an instrument for personal growth and for understanding interpersonal relationships, personal affections and social relationships are porous environments. Thus, in the present work, discussions in the religious group reactivated the account-making work compared to the previous failed marriage, even for those who embarked on new romantic relationships. Even the account-making activity relating to religious ostracism did not remain confined to the coping stage but continued over time, remaining always present in the participants’ thoughts and gaining further importance in the discussions of the new religious group.

The account-making activity present in the coping stage is also functionally different from the account-making that occurs even a long time after social death is experienced. The former can be referred to as ‘prospective account-making’, as it allows for the organization of activities aimed at maintaining inclusion. The second can be called ‘retrospective account-making’, as it helps in the process of the interpretation, acceptance and closure of a past event [63]. Ultimately, compared to short-lived and minor forms of ostracism, in the presence of long-lasting and severe ostracism within relationships that have involved a great deal of personal investment, it seems that not all psychological processes foreseen by the temporal need–threat model end once each stage is overcome. The coping stage’s own account-making continues over time within a reconstructive process of individual and social identity to make sense of one’s biography, not to face difficulties when they arise, but to avoid these difficulties from needing to be addressed again. Therefore, the experience of having been ostracized in significant relationships is not completely archived in the past—rather, it remains always available for periodic reinterpretation and revisiting.

The cumulative ostracism the participants in this research suffered presents an analogy with the cumulative trauma that occurs in intra-family abuse [64,65]. When a son or daughter is abused by a parent, the other parent is often a figure who ignores the victim’s psychological pain and rejects any request for help. Whatever the reasons for this distancing, for the victim, it is always suffering that adds up to suffering. In the present research, those who have suffered ostracism in marriage feel like victims who have sought support in other significant reference figures. Just as the child abused by his father seeks help in those who he believes can be comforting and helpful, so the partners in crisis in the couple’s relationship sought support from the priests, but they received further ostracism in return. The present research did not aim to investigate the reasons that can push religious and members of Catholic communities to ostracize separated people; it can be assumed that this behavior is inflicted more or less consciously to protect the rule of the indissolubility of marriage. With the separation, the commitment that the spouses had made to be faithful to one another and to be a ‘symbol’ for the Church is lost. When the promise is broken, and this sign is no longer visible to the Catholic community, uncertainty and disorientation can ensue. It can, therefore, be assumed that the ostracizing reaction does not intend to further injure those who already suffer from the broken marriage bond, but it intends to preserve certain values of the Church. This form of ostracism could be termed ‘defensive’ [26,27]. However, like the mother who ignores the request for help from her abused child so as not to endanger her marriage, putting certain values before the distress and psychological pain of others inevitably produces further psychological pain. The present participants shared the perception that the Catholic Church was too narrow-minded to include them as separated and/or divorced individuals. Such a perception recalls the notion of ‘entitativity’ [66], which indicates the feeling of being in front of an aggregate of people definable as a cohesive whole. This problem was well known to the coordinators of the House of Tenderness, who, in the preliminary research talks, underlined that one of their objectives was to make the participants in the group understand that they had suffered ostracism from ‘singular’ priests, and that these priests did not represent the whole Catholic Church. Social resurrection can happen beyond what was taken into account within the present study. Experiences such as that of the House of Tenderness are not common: some people come to these experiences randomly. Some people may never find a welcoming and caring context in which to experience re-inclusion and reconnection, and they may never experience social resurrection. The Berit group represents a resource that makes it possible to face the social suffering that affects these people.

## 5. Conclusions

Cumulative ostracism from the ‘marriage–religious group’ is a unique experience, one that this study has started to shed light on. The present work well illustrates and extends Williams’ ostracism theory [9,67] to the real-life context of a religious group of separated and divorced individuals. The results suggest that it is important to analyze the phenomenon of ostracism through the perspective of a mutual influence of different memberships on individual biography. The literature shows that re-inclusion after minor and short-term ostracism can lead to a complete recovery of belonging, sense of control, self-esteem and feelings of a significant existence [68]. This study has shown that re-inclusion is also possible following severe episodes of long-term ostracism, even if the scars can continuously reactivate psychological coping processes in the life cycle. The narratives included in the present study are limited by the personal experiences of the participants included in the present research, and thus possible alternative experiences remain open to investigation.

Ostracism has been carefully investigated in laboratory studies with a prevalent focus on the consequences for ostracized people. Although experimental studies may allow researchers to draw strong conclusions about the causality of the psychological processes involved, researchers generally design manipulations of ostracism in such a way that participants clearly recognize that they have been ostracized during their social interaction, which usually lasts a few minutes. However, these manipulations may not represent all the aspects through which ostracism occurs in particular social contexts. In addition, a still limited number of works has focused to date on understanding the sources of ostracism, on their possible interaction and peculiar dynamics in specific areas. We believe religious belonging is one of these areas. For this reason, the perspective adopted here can help to expand our understanding of ostracism as a real-life phenomenon.

## Figures and Tables

**Table 1:** Ostracism in Marriage.

Themes	Subthemes	Frequencies	
		Instances of Occurrence	% Across Participants
Immediate Stage	Psychological Pain and Anger	74	96
	Threat to Self-Esteem	63	88
	Threat to the Sense of Control	58	92
Coping Stage	Prospective Accounts on Marriage	36	84
	Causal and Responsibility Attributions	29	76
	Prayer/Search for Religious Support	69	92
Resignation Stage	Meaninglessness	51	72
	Impotence	34	80
	Alienation	26	64
Social Death	Loss of Individual Identity	53	92
	Loss of Partner Support	46	88
	Ruminations	41	56
	Explanations of Loss	68	76
	Development of ‘Irregular’ Romantic Relationships	21	48

**Table 2:** Ostracism in Religious Group.

Themes	Subthemes	Frequencies	
		Instances of Occurrence	% Across Participants
Immediate Stage	Psychological Pain	72	92
	Disbelief	57	84
	Intra-Group Discomfort	67	92
Coping Stage	Prospective Accounts on Religion	48	88
	Engaging in Religious Behaviors	29	76
	Seeking Support from Religious Figures	24	64
Resignation Stage	Distrust	47	84
	Disappointment	29	76
	Inadequacy	28	80
Social Death	Loss of Social Identity	45	88
	Loss of Group Support	56	92
Social Resurrection	Retrospective Accounts on Marriage and Religion	66	96
	Renewed Vision of Life	71	92
	Individual Growth	68	92
	Support to ‘Irregular’ Romantic Relationships	22	52
